# Clinical, Laboratory, and CT Morphological Factors Associated with 3-Month Functional Outcomes in Spontaneous Intracerebral Hemorrhage: A Retrospective Cohort Study

**DOI:** 10.3390/brainsci16070733

**Published:** 2026-07-11

**Authors:** Mustafa Harun Şahin, Özhan Özcan, Oğuzhan Kurşun

**Affiliations:** 1 Department of Neurology, Balikesir Ataturk City Hospital, 10100 Balikesir, Türkiye; 2 Department of Critical Care, Sinop Ataturk State Hospital, 57000 Sinop, Türkiye; ozhanturkey@hotmail.com; 3 Department of Neurology, Ankara Bilkent City Hospital, 06800 Ankara, Türkiye; ozikursun@hotmail.com

**Keywords:** spontaneous intracerebral hemorrhage, functional outcome, modified Rankin Scale, CT morphological markers, laboratory biomarkers, ICH score, FUNC score

## Abstract

Background/Objectives: Spontaneous intracerebral hemorrhage (ICH) is associated with high mortality and disability. We aimed to identify clinical, laboratory, and imaging factors independently associated with 3-month functional outcome across non-contrast computed tomography (NCCT) morphological markers, laboratory biomarkers, and established clinical scoring systems. Methods: This retrospective cohort study included 337 consecutive patients with spontaneous ICH admitted between 2019 and 2022. Admission clinical findings, laboratory parameters, NCCT morphological markers (swirl, blend, black hole, island, hypodensities, and satellite signs), volumetrically segmented hematoma volumes, and prognostic scores (ICH and FUNC scores) were recorded. Poor functional outcome was defined as modified Rankin Scale (mRS) 3–6 at 3 months. Independent predictors were identified using a primary Firth penalized multivariable logistic regression model; discrimination (area under the ROC curve, AUC), calibration, and optimism-corrected performance (1000-resample bootstrap) were assessed, and incremental value over the ICH and FUNC scores was tested with the DeLong method. Results: Poor functional outcome occurred in 60.2% of patients, and 3-month mortality was 36.8%. In the primary penalized model, independent predictors of poor outcome were lower admission Glasgow Coma Scale, larger hematoma volume, NCCT hypodensities, history of hypertension, and lower low-density lipoprotein (LDL) cholesterol. The model showed good discrimination (apparent AUC 0.881; optimism-corrected 0.872) and calibration (slope 0.92) and significantly outperformed the ICH and FUNC scores (DeLong *p* = 0.009 and *p* < 0.001, respectively). Several laboratory and treatment variables reached significance only in an unpenalized model and were statistically unstable; these were treated as exploratory. Although swirl, blend, black hole, and island signs were associated with poor outcomes in univariable analysis, they did not remain independent predictors in the final model. The ICH and FUNC scores were significantly associated with poor functional outcome in univariable analysis. Conclusions: Poor 3-month functional outcome after spontaneous ICH is independently associated with neurological severity, hematoma volume, NCCT hypodensities, and selected clinical and laboratory factors. Integrating clinical, volumetric, and CT morphological parameters improved discrimination over established prognostic scores; however, these findings are associative and require prospective, multicenter validation before clinical implementation.

## 1. Introduction

Intracerebral hemorrhage (ICH) is the second most common stroke subtype after ischemic stroke and a major cause of death and long-term disability. Spontaneous ICH accounts for approximately 9–27% of all strokes worldwide, with early mortality consistently reported at 30–40% [1,2,3,4].

ICH is a neurological emergency: ongoing bleeding and secondary injury can drive hematoma expansion, progressive deterioration, persistent disability, and death [5]. Non-contrast computed tomography (NCCT) is the first-line diagnostic modality, valued for its speed, availability, and high accuracy in the hyperacute setting [6,7].

Hematoma volume and hemorrhage burden are among the strongest determinants of functional outcome and are closely linked to neurological worsening [8,9]. Prognosis is further shaped by patient risk factors and comorbidities, etiology, hemorrhage location (e.g., lobar vs. deep; supratentorial vs. infratentorial), initial burden, and intraventricular extension [10,11,12,13,14]. While all patients require evidence-based acute care, recognizing high-risk features can guide more intensive monitoring and tailored management.

Prognostic determinants in ICH span three domains: imaging markers, blood-based biomarkers, and clinical scoring systems [15]. Imaging markers include hemorrhage location and volume, intraventricular hemorrhage (IVH), and NCCT/CT angiography (CTA) features of established prognostic value—the spot sign on CTA and NCCT signs such as blend, black hole, island, hypodensities, and swirl [6,16,17,18]. Blood-based biomarkers reflect systemic stress, inflammation, and coagulation, including glucose, inflammatory indices (white blood cell count, neutrophils, and neutrophil-to-lymphocyte ratio), and coagulation parameters [19,20,21]. The ICH and FUNC scores integrate clinical and radiological variables to estimate mortality and functional outcome [12,14].

Against this background, in the present study, we sought to address a critical gap: whereas previous researchers have largely evaluated clinical, imaging, and laboratory predictors in isolation, few have subjected all three prognostic domains to simultaneous multivariable analysis within a well-defined, consecutive cohort. We therefore aimed to identify independent predictors of 3-month functional outcome across clinical severity, quantitative hemorrhage burden, CT morphological markers, laboratory biomarkers, and validated prognostic scores; to determine which of these markers retain prognostic value after mutual adjustment; and to characterize the clinical phenotype most strongly associated with unfavorable recovery. In addition, we explored determinants of length of hospital stay to better describe the clinical course in this cohort. Because these domains are mutually correlated—clinical severity, hemorrhage volume, and systemic biomarkers frequently co-vary—associations observed within a single domain may not persist once the others are accounted for; a simultaneous, adjusted analysis is therefore required to distinguish genuinely independent prognostic markers from those whose apparent effect is mediated by hemorrhage severity.

## 2. Materials and Methods

### 2.1. Study Design, Setting, and Ethical Approval

This retrospective cohort study was conducted at Ankara City Hospital and approved by the Ankara City Hospital No. 1 Clinical Research Ethics Committee (9 February 2022; approval no: E1-22-2383). The study was performed in accordance with the Declaration of Helsinki.

### 2.2. Study Population

We screened all adult patients (≥18 years) who presented to the Ankara City Hospital emergency department between March 2019 and June 2022 and were diagnosed with spontaneous ICH requiring hospitalization in the neurology ward and/or intensive care unit. No upper age limit was applied.

Electronic hospital records were searched using diagnostic codes related to intracerebral hemorrhage, cerebral bleeding, and cerebrovascular events, yielding 8915 records. After detailed chart review, 344 patients met the diagnostic criteria for spontaneous ICH. Following the application of exclusion criteria, the final cohort consisted of 337 patients.

### 2.3. Exclusion Criteria

Patients were excluded if they had

- Isolated intraventricular hemorrhage without accompanying intraparenchymal hematoma (*n* = 7);
- Traumatic intracerebral hemorrhage;
- Hemorrhage secondary to a structural lesion (e.g., intracranial tumor), vasculitis, or hemorrhagic transformation of ischemic stroke;
- Missing baseline neuroimaging or insufficient clinical data to define the primary outcome at 3 months.

### 2.4. Data Collection

Data were extracted from electronic medical records and included the following:

- Demographics: age and sex.
- Medical history and risk factors: diabetes mellitus, hypertension, coronary artery disease/cardiac disease, prior ischemic stroke, prior intracranial hemorrhage, malignancy, epilepsy, dementia, chronic kidney disease, chronic pulmonary disease, hypothyroidism, and Parkinson’s disease.
- Antithrombotic therapy: antiplatelet use, anticoagulant use, and combined use.
- Clinical parameters at admission: length of hospital stay, electrocardiography (ECG) findings, admission systolic and diastolic blood pressure (when numerical values were unavailable but hypertension was documented, this was recorded separately), and admission Glasgow Coma Scale (GCS) score.
- Laboratory parameters at admission: glucose, estimated glomerular filtration rate (eGFR), urea, creatinine, calcium, sodium, potassium, chloride, prothrombin time, international normalized ratio (INR), activated partial thromboplastin time (aPTT), C-reactive protein (CRP), white blood cell count (WBC), neutrophil, eosinophil, and lymphocyte counts, neutrophil-to-lymphocyte ratio (NLR), platelet count, mean platelet volume (MPV), hemoglobin, lipid profile (LDL, HDL, total cholesterol, and triglycerides), alanine aminotransferase (ALT), aspartate aminotransferase (AST), lactate dehydrogenase (LDH), creatine kinase (CK), and uric acid.

### 2.5. Imaging Evaluation and CT Markers

All patients underwent NCCT at admission; CTA was performed when clinically indicated. Baseline NCCT/CTA images were independently reviewed by two investigators blinded to clinical outcomes; disagreements were resolved by consensus. Inter-rater agreement was excellent for all imaging markers, with Cohen’s kappa values ranging from 0.87 to 0.99 (spot sign κ = 0.88; blend sign κ = 0.90; black hole sign κ = 0.96; island sign κ = 0.97; hypodensities κ = 0.95; swirl sign κ = 0.87; satellite sign κ = 0.90), as well as for clinical scores (GCS κ = 0.98; ICH score κ = 0.98; 3-month mRS κ = 0.98; FUNC score κ = 0.99).

Hemorrhage location was first classified as supratentorial or infratentorial, and then categorized anatomically (frontal, parietal, occipital, temporal, caudate, putaminocapsular, thalamic, cerebellar, mesencephalic, pontine, or medullary). The presence of intraventricular hemorrhage (IVH) was recorded.

**Figure 1:**
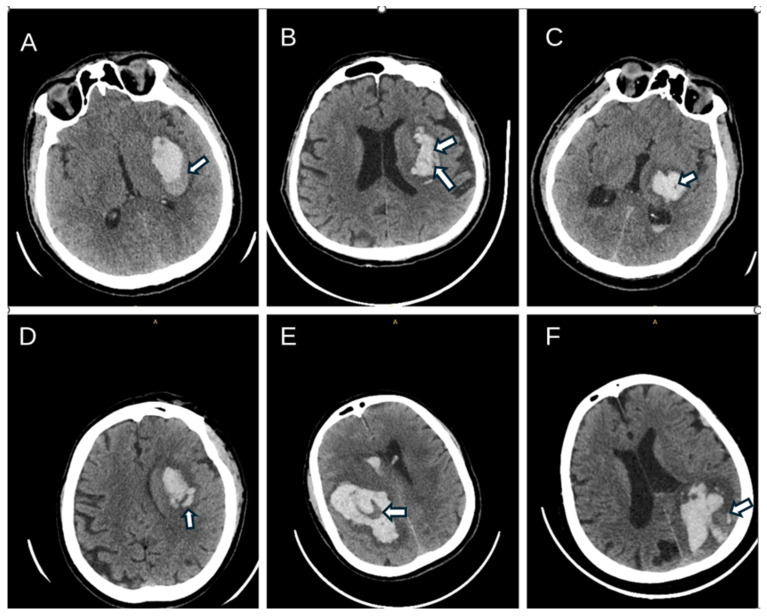
Representative non-contrast cranial CT images demonstrating markers of hematoma instability associated with functional outcome in intracerebral hemorrhage. (**A**) Blend sign, (**B**) intrahematomal hypodensities, (**C**) black hole sign, (**D**) island sign, (**E**) swirl sign, and (**F**) satellite sign.

The following imaging prognostic markers were assessed on NCCT/CTA based on established definitions: spot sign, blend sign, black hole sign, island sign, hypodensities, swirl sign, and satellite sign (Figure 1). Spot sign was evaluated on CTA only; because CTA was not performed in all patients, spot sign was not included in the outcome analyses. The time from symptom onset to NCCT was not systematically recorded in the electronic medical records and could not be reliably extracted for all patients; this represents a limitation acknowledged in Section 4.6.

### 2.6. Hematoma Volume Measurement

Intraparenchymal and intraventricular hematoma volumes were measured on baseline NCCT using the AW VolumeShare 7 workstation (GE Medical Systems SCS, Yvelines, France) with volume viewer/hematoma segmentation software. The automated segmentation algorithm identifies hematoma boundaries using a Hounsfield unit (HU) threshold-based approach (typically 40–80 HU for acute hemorrhage), which delineates hyperdense hemorrhagic regions from surrounding brain parenchyma on a slice-by-slice basis and reconstructs a three-dimensional volumetric estimate. When automatic segmentation was inadequate—due to irregular hematoma morphology, proximity to bone, or suboptimal image quality—manual region-of-interest (ROI) delineation was performed by the reviewing investigator. All segmentations were visually verified by a second investigator; discrepancies were resolved by consensus. Total hematoma burden was evaluated by quantifying intraparenchymal volume and IVH volume; when appropriate, a combined total volume was also reported.

### 2.7. Outcomes and Prognostic Scoring Systems

Functional outcome was assessed using the modified Rankin Scale (mRS) at admission (reflecting acute presenting neurological status rather than premorbid function), at discharge, and at 3 months. Three-month mRS scores were obtained from outpatient clinic records; for patients without hospital follow-up, external records were accessed via the national e-health system (e-Pulse). Poor functional outcome was defined as a modified Rankin Scale (mRS) score of 3–6 at 3 months.

To evaluate disease severity, mortality risk, and functional prognosis, the following prognostic scores were calculated from admission clinical and imaging data:

- ICH score: It incorporates age, admission GCS score, hematoma volume, IVH presence, and infratentorial location, and was used to estimate early mortality risk and overall severity [12].
- FUNC score: It incorporates age, admission GCS score, hematoma volume, hemorrhage location, and IVH presence to estimate the likelihood of functional independence at 3 months [14].

### 2.8. Statistical Analysis

Statistical analyses were performed using IBM SPSS Statistics version 23.0 (IBM Corp., Armonk, NY, USA). Normality was assessed using the Kolmogorov–Smirnov test. Categorical variables were compared using the chi-square test, Yates’ correction, or Fisher’s exact test, as appropriate, with Bonferroni-adjusted z-tests for multiple comparisons. Continuous variables were compared using the independent samples *t*-test for normally distributed data and the Mann–Whitney U test for non-normally distributed data.

**Figure 2:**
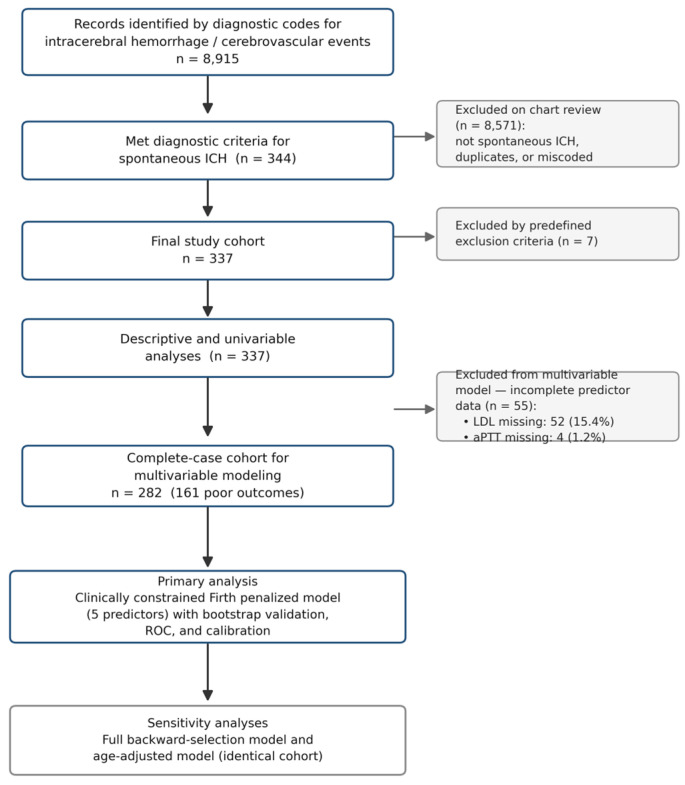
STROBE flow diagram of patient identification, selection, and the analysis cohort. Of 8915 screened records, 344 met criteria for spontaneous ICH and 337 formed the final cohort; the reduction to 282 patients for multivariable modeling was driven predominantly by missing LDL cholesterol (15.4%). Boxes along the central vertical path denote the patients included at each stage; the gray boxes to the right indicate the numbers excluded and the reasons for exclusion.

Linear regression analysis was used to identify factors associated with length of hospital stay; the length-of-stay variable was log-transformed (natural logarithm) prior to analysis to satisfy normality assumptions, as confirmed by the Kolmogorov–Smirnov test. Predictors of poor functional outcome were first explored using univariable binary logistic regression analysis. The primary objective of this analysis was explanatory—to identify factors independently associated with 3-month functional outcome—rather than to derive or validate a deployable clinical prediction model. Measures of predictive performance (discrimination and calibration) are therefore reported as a secondary, supportive assessment of how well the identified factors jointly separate outcomes, and not as validation of a prediction tool intended for clinical use. For the multivariable analysis, a clinically constrained primary model was defined for the revised analysis on the basis of prior evidence and clinical relevance, comprising admission GCS, history of hypertension, intraparenchymal hematoma volume, NCCT hypodensities, and LDL cholesterol, and was estimated using Firth penalized logistic regression to reduce small-sample bias and to accommodate quasi- or complete separation among sparse imaging markers [22,23]. The composite ICH and FUNC scores were excluded from the multivariable model to avoid tautological overlap with their constituents and were instead retained as external comparators; individual clinically relevant variables, such as admission GCS and hematoma volume, were retained as candidate predictors. Age was not entered as a separate predictor because it is a component of both composite scores and its effect is largely captured by admission GCS and hematoma volume; in a sensitivity analysis, adding age left the other estimates essentially unchanged (age adjusted OR 1.03 per year; *p* = 0.007; AUC 0.889). To limit type I error arising from the large number of laboratory variables screened univariably, associations were additionally evaluated with Benjamini–Hochberg false discovery rate correction. Model discrimination was quantified by the AUC and internally validated using 1000 bootstrap resamples to obtain optimism-corrected estimates; calibration was assessed by the calibration slope and plot [24,25]. The incremental value of the multimodal model over the ICH score, the FUNC score, and admission GCS alone was compared using the DeLong test. Multivariable analyses were performed on the 282 patients with complete data for all candidate predictors, ensuring that the primary model and the sensitivity analysis were directly comparable on an identical cohort. Reporting follows the TRIPOD statement [26]. As a sensitivity analysis, the previously used automated model—entering univariably significant and clinically relevant variables into a backward Wald selection—was retained (Table 6). Variables such as glucose and CRP, together with inflammatory markers including NLR, did not retain independent prognostic value after adjustment for stronger predictors such as admission GCS and hematoma volume, reflecting multicollinearity and mediation through these dominant covariates rather than an absence of biological relevance. Logistic regression results are presented as odds ratios (ORs) with 95% confidence intervals (CIs). A two-sided *p* value < 0.05 was considered statistically significant. Firth penalized regression, bootstrap internal validation, calibration, and ROC/DeLong comparisons were performed in R version 4.3 (R Foundation for Statistical Computing, Vienna, Austria) using the logistf, rms, and pROC packages; descriptive and univariable analyses used IBM SPSS Statistics version 23.0. The derivation of the analysis cohort is summarized in Figure 2.

## 3. Results

### 3.1. Baseline Characteristics of the Study Population

Baseline clinical and radiological characteristics are summarized in Table 1, with characteristics stratified by 3-month functional outcome presented in Table 2. The cohort comprised predominantly older adults, mostly male, with hypertension as the leading comorbidity. Intraventricular hemorrhage was common, and most hemorrhages were supratentorial. Admission severity and radiological burden varied widely, and 3-month outcomes were generally unfavorable.

The median age was 68 years (range 20–96), and 59.3% of patients were male. The median admission GCS was 12 (range 3–15), and hypertension was the most prevalent comorbidity (68.0%). For antithrombotic therapy, 52.8% used no agent, 30.0% antiplatelet only, 14.2% anticoagulant only, and 3.0% combined therapy. The median hematoma volume was 13.30 mL (range 0.1–164), and IVH was present in 124 patients (36.8%), with a median IVH volume of 7.96 mL (IQR 2.46–18.90; range 0.10–131.14). Most hemorrhages were supratentorial (87.5%), and the median hospital stay was 14 days. At 3 months, the median mRS was 4; 60.2% of patients had poor outcome (mRS 3–6) and 3-month mortality was 36.8%.

### 3.2. Association Between Non-Contrast CT Imaging Markers and Poor Functional Outcome

Associations between NCCT imaging markers and outcome are shown in Table 3. Several markers were more frequent among patients with poor outcome. The swirl sign was present in 6.5% and showed complete separation: all 22 swirl-positive patients (100%) had poor outcome. Because none had a favorable outcome, a stable odds ratio could not be estimated; a Haldane–Anscombe-corrected value (OR 33.35, 95% CI 2.00–554.64) is provided for description only. Blend sign (7.7%; OR 8.85; *p* = 0.001), black hole sign (10.7%; OR 13.28; *p* < 0.001), island sign (21.4%; OR 6.25; *p* < 0.001), and hypodensities (18.4%; OR 8.13; *p* < 0.001) were also significantly associated with poor outcome, whereas the satellite sign (4.2%) was not (OR 4.15; *p* = 0.087).

### 3.3. Prognostic Scores and Functional Outcome

Associations between prognostic scores and outcome are detailed in Table 4. Patients with poor outcome had higher ICH scores (median 2, range 0–6) than those with favorable outcome (median 1, range 0–3; OR 4.14, 95% CI 3.04–5.64; *p* < 0.001) and lower FUNC scores (median 8, range 1–11 vs. median 10, range 7–11; OR 0.47, 95% CI 0.39–0.57; *p* < 0.001). Admission GCS was also markedly lower with poor outcome (median 9, range 3–15 vs. median 14, range 3–15; OR 0.60, 95% CI 0.53–0.68; *p* < 0.001).

### 3.4. Independent Predictors of Poor Functional Outcome

Independent predictors of poor functional outcome in the primary penalized model are presented in Table 5. Lower admission GCS (adjusted OR 0.66, 95% CI 0.57–0.74), larger hematoma volume (OR 1.04 per mL, 95% CI 1.01–1.06), NCCT hypodensities (OR 3.89, 95% CI 1.46–11.65), history of hypertension (OR 2.79, 95% CI 1.37–5.95), and lower LDL cholesterol (OR 0.88 per 10 mg/dL, 95% CI 0.80–0.97) were each independently associated with poor outcome; all five estimates were stable, with no evidence of separation. Several NCCT markers that were significant in univariable analysis (swirl, blend, black hole, and island signs) did not retain independence after adjustment. Three additional variables—prolonged aPTT, higher hemoglobin, and combined antiplatelet–anticoagulant use—attained significance only in the unpenalized automated backward-selection model (Table 6) but were statistically unstable: aPTT had no univariable association (*p* = 0.69) and lost significance when five extreme values were excluded; hemoglobin was non-significant univariably (*p* = 0.42), consistent with a suppression artifact; and combined therapy was based on only 10 patients with an implausibly wide confidence interval. These were therefore interpreted as exploratory and are not featured as robust predictors. Among the remaining laboratory parameters evaluated—including calcium, sodium, potassium, urea, creatinine, ALT, AST, LDH, CK, uric acid, MPV, HDL, and triglycerides—none showed a statistically significant association with poor functional outcome in univariable analysis. After Benjamini–Hochberg correction for multiple comparisons across the laboratory panel, only CRP, glucose, LDH, and LDL cholesterol remained significantly associated with poor functional outcome.

### 3.5. Model Performance and Incremental Value

**Figure 3:**
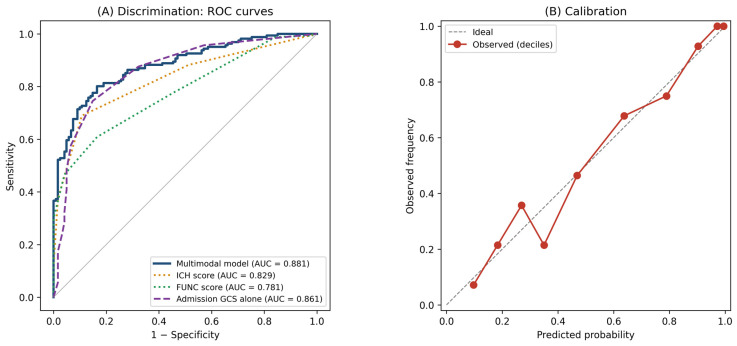
Performance of the primary multimodal model. (**A**) Receiver operating characteristic curves for the multimodal model versus the ICH and FUNC scores. (**B**) Calibration plot showing observed versus predicted probability of poor outcome by decile of predicted risk.

The primary penalized model showed good discrimination, with an apparent AUC of 0.881 (Figure 3A). Internal validation using 1000 bootstrap resamples indicated minimal optimism (0.009), yielding an optimism-corrected AUC of 0.872, consistent with limited overfitting despite the multivariable structure (161 events for five predictors). Calibration was good, with an optimism-corrected calibration slope of 0.92 and close agreement between predicted and observed risk across the range of predicted probabilities (Figure 3B). In head-to-head comparisons on the identical cohort, the multimodal model discriminated significantly better than the ICH score (AUC 0.881 vs. 0.829; DeLong *p* = 0.009) and the FUNC score (0.881 vs. 0.781; *p* < 0.001). Discrimination was numerically higher than admission GCS alone (0.881 vs. 0.861), but this difference did not reach statistical significance (*p* = 0.17), indicating that most—though not all—of the model’s discriminatory advantage over established scores is anchored by baseline neurological severity. The full automated backward-selection model, which additionally retained aPTT, hemoglobin, and combined antithrombotic therapy, is provided for transparency as a sensitivity analysis (Table 6); its discrimination was similar (AUC 0.894), confirming that the excluded variables contributed negligibly to model performance.

In a sensitivity analysis adding age to the primary model, age was independently associated with poor outcome (adjusted OR 1.03 per year, 95% CI 1.01–1.06; *p* = 0.007), while the other estimates were essentially unchanged (admission GCS OR 0.66, hypertension OR 2.23, hematoma volume OR 1.04, hypodensities OR 3.89, and LDL OR 0.99 per mg/dL) and discrimination was similar (AUC 0.889). Age was nevertheless excluded from the primary model because it is a constituent of both the ICH and FUNC scores used as external comparators; its prognostic information is thus already represented in those benchmarks.

### 3.6. Factors Associated with Length of Hospital Stay

Table 7 presents the linear regression for factors associated with length of hospital stay (log-transformed for normality). The overall model was significant (F = 6.662; *p* < 0.001; R^2^ = 0.197; adjusted R^2^ = 0.167), with variance inflation factors of 1.037–1.276 indicating no multicollinearity. Higher admission mRS (B = 0.272; *p* < 0.001), elevated NLR (B = 0.024; *p* = 0.048), and epileptic seizures (B = 0.465; *p* = 0.035) were independently associated with longer stay, whereas greater hematoma volume (B = −0.007; *p* = 0.046) and the swirl sign (B = −0.653; *p* = 0.033) were associated with shorter stay. Because early mortality strongly influences length of stay, these associations are considered exploratory; death is a competing event, and the shorter stays associated with larger hematomas and the swirl sign most likely reflect early death rather than faster recovery.

## 4. Discussion

In this single-center cohort of 337 patients with spontaneous ICH, poor 3-month functional outcome (mRS 3–6) was common, reflecting the high morbidity and mortality of ICH. Consistent with prior work, baseline neurological severity and hemorrhage burden were the dominant determinants: older age, lower admission GCS, larger volumetrically measured hematomas, and IVH were all strongly associated with poor recovery.

Beyond these established predictors, NCCT markers of hematoma instability—swirl, blend, black hole, island signs and hypodensities—were more frequent among patients with poor outcome, though only hypodensities remained independent on multivariable analysis. Established tools also performed well: the ICH score provided robust risk stratification, and the FUNC score added complementary information on functional prognosis.

Together, these findings support integrating clinical severity, quantitative hemorrhage burden, imaging phenotype, and validated scores for early prognostication, which may improve risk stratification and acute decision-making.

### 4.1. Clinical Severity and Hemorrhage Burden

Despite a lower incidence than ischemic stroke, ICH carries high mortality and substantial long-term disability [1,3,4,27]. In our cohort, poor 3-month outcome was common, emphasizing the importance of early prognostic assessment.

Consistent with the prior literature, baseline neurological severity and hemorrhage burden were the most important determinants of outcome, with admission GCS strongly and inversely associated with poor outcome, in keeping with its role in established prognostic models [12,14].

Hematoma volume was also a major prognostic determinant. Notably, we measured volume with dedicated volumetric segmentation software rather than the ABC/2 method [28]; although ABC/2 is a practical bedside tool, segmentation is more precise, particularly for irregular hematomas. The strong volume–outcome association in our cohort reinforces the central role of hemorrhage burden in prognosis.

IVH is a well-established adverse marker and a component of the ICH score [12,13]. Both IVH presence and volume were associated with worse outcome univariably (IVH volume OR 1.15 per mL; *p* < 0.001), but neither remained independent after adjustment for admission GCS and hematoma volume, likely because ventricular blood burden is closely correlated with these dominant predictors. Quantitative IVH assessment may warrant further study, though our data do not establish an independent dose-dependent effect.

### 4.2. CT Morphological Markers and Functional Outcome Prediction

NCCT markers of hematoma instability are increasingly studied as outcome predictors [5,6]. In our cohort, swirl, blend, black hole, and island signs and hypodensities were each associated with poor outcome univariably, but only hypodensities remained independent in the final model.

The swirl sign showed a particularly strong association with poor outcome, exceeding even the 61% one-month mortality reported by Selariu et al. [29]. Its absence from the final model reflects complete separation: every swirl-positive patient had a poor outcome, so logistic regression cannot estimate a finite coefficient. This is a statistical artifact, not a lack of clinical importance—it reflects the near-certain unfavorable course of these patients. The sign’s variance is absorbed by hematoma volume and admission GCS, since swirl-positive patients typically present with larger hematomas and greater neurological deterioration. Pathophysiologically, the swirl sign indicates active, unclotted bleeding driving rapid expansion, and the shorter hospital stays in this subgroup are consistent with early mortality.

Li et al. reported a blend sign prevalence of 16% with reliable prediction of poor outcome [16]. We observed it in 7.7% (univariable OR 8.8), though it lost independence after adjustment; the lower prevalence may reflect differences in imaging timing or patient selection.

Black hole (10.7%) and island sign (21.4%) prevalences were comparable to the 13.9% and 26.4% reported by Sporns et al. [18], and both were significantly associated with poor outcome initially. The satellite sign’s lack of significance likely reflects its low prevalence (4.2%) and limited statistical power.

### 4.3. Prognostic Scoring Systems

The ICH score remains one of the most widely validated and clinically used severity grading systems in spontaneous ICH [12]. In our cohort, increasing ICH score was strongly associated with poor functional outcome. In the present analysis, the multimodal model discriminated significantly better than the ICH score (AUC 0.881 vs. 0.829; DeLong *p* = 0.009), supporting the added value of integrating imaging and laboratory information beyond established severity grading, although the ICH score alone still provided strong stand-alone discrimination. Given its simplicity and bedside applicability, the ICH score continues to serve as a reliable primary risk stratification tool in clinical practice.

The FUNC score, designed to predict 90-day functional independence rather than early mortality [14], was also significantly associated with outcome. Combining the ICH and FUNC scores may therefore provide complementary information on early severity and long-term recovery potential.

Admission GCS correlated strongly and inversely with poor outcome, consistent with its inclusion in most prognostic scores [12,30]. Higher admission mRS was associated with longer hospital stay, reflecting greater care needs.

### 4.4. Systemic and Treatment-Related Factors

Hypertension was an independent predictor of poor outcome, consistent with its central role in ICH pathophysiology [31,32,33,34]. Although admission blood pressure was higher in patients with unfavorable outcomes, these values should be interpreted cautiously, as acute stress and early treatment can affect initial readings.

Antiplatelet and anticoagulant use are recognized risk factors for ICH and poor outcome [21,35]. Only 10 patients (3.0%) received combined therapy (one favorable vs. nine poor). Although the adjusted estimate suggested markedly increased risk (adjusted OR 22.8, 95% CI 1.9–270.3), it derived from this very small subgroup and was highly unstable; it should be regarded as hypothesis-generating. Its direction is nonetheless consistent with the elevated bleeding risk and larger hematomas associated with combined regimens.

Hyperglycemia was significantly associated with poor outcome univariably, consistent with INTERACT2, which identified hyperglycemia and diabetes as independent markers of poor outcome in mild-to-moderate ICH [36].

Unlike studies suggesting that reduced eGFR is a risk factor for ICH [37] and poor outcome [38], we found no significant association between eGFR, urea, or creatinine and outcome.

Elevated CRP was significantly associated with poor outcome (*p* = 0.002), consistent with Napoli et al., who found that CRP > 10 mg/L predicts early deterioration and poor outcome [20]. In univariable logistic regression, NLR showed a modest association with poor functional outcome (OR 1.04 per unit; *p* = 0.030), although the between-group difference in NLR did not reach statistical significance on Mann–Whitney U testing (Table 2; *p* = 0.083). After adjustment for admission GCS and hematoma volume, neither marker retained independent prognostic value. This fits the known relationship between ICH severity and systemic inflammation: patients with larger hematomas and lower GCS mount a greater inflammatory response, so CRP and NLR act partly as mediators rather than independent drivers. Once GCS and hematoma volume entered the model, these markers added no independent explanatory power—attenuation that reflects shared variance rather than an absence of biological relevance, in line with studies of NLR’s prognostic value in ICH [19,39].

Higher LDL and total cholesterol were protective against poor outcome. This paradoxical effect is well documented in systematic reviews and meta-analyses [40], possibly reflecting cholesterol’s role in vascular integrity or as a marker of better nutritional status.

Hemoglobin was examined as a candidate predictor but was not associated with poor outcome univariably (*p* = 0.42); the inverse direction seen after adjustment most likely reflects a suppression effect rather than a true biological relationship. This contrasts with Kuramatsu et al., who identified anemia as an independent predictor [41]. Given its lack of univariable association, hemoglobin is unlikely to be a robust predictor and may represent a statistical artifact.

Linear regression identified admission mRS and NLR as factors associated with longer stay, reflecting greater disability and inflammatory burden. Paradoxically, larger hematoma volume and the swirl sign were associated with shorter stay, likely due to early mortality, while absence of seizures also predicted shorter stay, as seizures can complicate the clinical course.

### 4.5. Incremental Value of the Present Study

This study makes three contributions. First, hemorrhage burden was quantified with dedicated volumetric segmentation for both intraparenchymal and intraventricular components rather than the ABC/2 approximation, enabling more precise characterization of total hemorrhagic load. Although IVH volume was strongly associated with outcome univariably, it did not remain independent after adjustment for admission GCS and hematoma volume, consistent with its close link to overall burden; volumetric quantification nonetheless offers a more granular measure that warrants further study.

Second, to our knowledge, this is among the first studies to formally document complete separation of the swirl sign with respect to 3-month outcome in a large consecutive cohort: every swirl-positive patient had a poor outcome (mRS 3–6). Although this precluded stable coefficient estimation, the clinical implication is clear—the swirl sign identified a very high-risk subgroup characterized by active bleeding and rapid expansion, consistent with the paradoxically shorter stays observed. However, given the small number of swirl-positive patients (*n* = 22) and the absence of imaging-timing data, this finding requires prospective validation before it can be used to guide monitoring or treatment decisions.

Third, by simultaneously entering clinical, laboratory, and CT morphological predictors into a unified framework, we determined which markers retain independent prognostic value after mutual adjustment—an approach that most prior single-domain studies cannot address. Among the CT markers evaluated, only hypodensities survived full adjustment, indicating that this feature captures prognostic information not fully explained by hematoma volume or neurological severity. Among laboratory parameters, low LDL cholesterol emerged as a stable independent contributor, underscoring the role of vascular biology in determining recovery; by contrast, prolonged aPTT reached significance only in the unpenalized model and was shown to be driven by a few extreme values, and was therefore not retained as a robust predictor. Notably, CRP and NLR—while associated with poor outcome in univariable logistic regression—did not retain independence after adjustment, reflecting mediation of their prognostic signal through admission GCS and hematoma volume rather than an absence of biological relevance. The association between combined antiplatelet–anticoagulant therapy and poor outcome, based on only 10 patients and yielding an unstable estimate, is reported as hypothesis-generating and merits confirmation in larger cohorts. In this sense, the independent contribution of hypodensities and the attenuation of inflammatory markers (CRP and NLR) after adjustment are confirmatory of prior reports, whereas the complete outcome separation of the swirl sign is, to our knowledge, a novel observation specific to 3-month functional prognosis.

Taken together, these contributions support a shift from single-domain to integrated multimodal prognostic assessment in spontaneous ICH. In this cohort, the multimodal model was well calibrated and significantly more discriminating than the ICH and FUNC scores (DeLong *p* = 0.009 and *p* < 0.001), although its advantage over admission GCS alone was numerical rather than statistically significant (*p* = 0.17), indicating that baseline neurological severity carries much of the prognostic information. Routine admission NCCT, standard laboratory panels, and validated clinical scoring systems are universally available tools; our data suggest that combining these domains improves discrimination over established scores, though the incremental value beyond GCS alone requires confirmation and prospective external validation before clinical use. Such an integrated strategy may improve early risk stratification and help identify patients who may benefit from more intensive monitoring or escalated care protocols.

### 4.6. Strengths and Limitations

Strengths of this study include the relatively large consecutive cohort, the standardized volumetric measurement of hematoma and IVH, the comprehensive evaluation of NCCT markers, and the integration of clinical, imaging, and laboratory domains within unified regression models.

Limitations include the retrospective design, single-center setting, potential interobserver variability in imaging interpretation, incomplete systematic CTA evaluation, and follow-up limited to 3 months. A key limitation is that the interval between symptom onset and NCCT was not systematically recorded. Because several NCCT morphological markers are time-dependent, variation in imaging timing may have influenced their detection and prevalence and could therefore have biased the reported associations between these markers and functional outcome. This interval should be prospectively documented and, where possible, adjusted for in future studies. Because the source images could not be re-accessed at the time of revision, a direct comparison between volumetric segmentation and the ABC/2 method was not feasible and remains a limitation to be addressed prospectively. Numerous laboratory variables were screened in univariable analysis; to mitigate multiplicity, we applied Benjamini–Hochberg false discovery rate correction, after which CRP, glucose, LDH, and LDL remained significant, but isolated associations should still be confirmed independently. Laboratory data were incomplete in a minority of patients (lipid profile missing in approximately 16%); multivariable models were fitted on complete cases (*n* = 282) without imputation, which may introduce selection bias. A complete-case analysis was preferred over multiple imputation because the missing-at-random assumption could not be verified, because imputing outcome-associated values for approximately 16% of patients could introduce additional bias given the limited number of events, and because complete-case analysis keeps the results transparent and directly interpretable; the consistency of estimates across the primary and sensitivity models argues against substantial distortion, although residual selection bias cannot be fully excluded. To address concerns regarding model stability and multiplicity, the primary analysis used a clinically constrained, penalization-based model with composite scores excluded and internal validation, whereas the earlier automated backward-selection model—which simultaneously included composite scores and several constituents—is now presented only as a transparency-oriented sensitivity analysis (Table 6). Finally, although discrimination, calibration, and optimism-corrected internal validation were assessed, the model has not undergone external validation; accordingly, our findings should be interpreted as associative and require prospective multicenter validation before clinical application. The inverse association between LDL cholesterol and poor outcome should be interpreted cautiously, as we did not adjust for statin use, nutritional status, or acute-phase effects, any of which may confound this relationship.

## 5. Conclusions

In this consecutive cohort of 337 patients with spontaneous ICH, 3-month functional outcome was independently associated with a convergent set of clinical, imaging, and laboratory factors. Admission neurological severity and volumetrically measured hematoma burden remained the dominant prognostic drivers. Among CT morphological markers, hypodensities independently predicted poor outcomes, while the swirl sign showed complete functional outcome separation—an observation that, although based on a small subgroup (*n* = 22) and not retained in the primary penalized model, may represent an early signal of a high-risk hemorrhage phenotype warranting prospective confirmation. Quantitative IVH assessment showed a strong univariable association with outcome that did not persist after adjustment for clinical severity and hematoma volume. Among laboratory parameters, lower LDL cholesterol was a stable independent correlate of poor outcome, whereas aPTT, hemoglobin, and combined antithrombotic therapy were unstable and are reported as exploratory only. The primary multimodal model was well calibrated and discriminated significantly better than established prognostic scores (optimism-corrected AUC 0.872). These findings reinforce the value of a multimodal prognostic framework integrating clinical severity, quantitative hemorrhage burden, CT morphology, and laboratory biomarkers at admission; because the analysis was exploratory and single-center, prospective external validation is required before the approach can inform individualized risk stratification in acute spontaneous ICH.

## Figures and Tables

**Table 1:** Baseline clinical and radiological characteristics of the study population.

Variable	Overall (*n* = 337)
Age, years, median (min–max)	68 (20–96)
Sex, male, *n* (%)	200 (59.3)
Admission Glasgow Coma Scale, median (min–max)	12 (3–15)
History of hypertension, *n* (%)	229 (68.0)
Antithrombotic therapy, *n* (%)	
None	178 (52.8)
Antiplatelet only	101 (30.0)
Anticoagulant only	48 (14.2)
Combined antiplatelet + anticoagulant	10 (3.0)
Hematoma volume, mL, median (min–max)	13.30 (0.1–164)
Intraventricular hemorrhage (IVH), *n* (%)	124 (36.8)
IVH volume (in patients with IVH), mL, median (min–max)	7.96 (0.10–131.14)
Hemorrhage location, *n* (%)	
Supratentorial	295 (87.5)
Infratentorial	42 (12.5)
Length of hospital stay, days, median (min–max)	14 (1–155)
Modified Rankin Scale at 3 months, median (min–max)	4 (0–6)
Poor functional outcome (mRS 3–6), *n* (%)	203 (60.2)
Mortality at 3 months, *n* (%)	124 (36.8)

Data are presented as median (minimum–maximum) for continuous variables and as number (percentage) for categorical variables. IVH volume is reported among the 124 patients with IVH. Poor functional outcome is defined as a modified Rankin Scale (mRS) score of 3–6. Abbreviations: IVH, intraventricular hemorrhage; mRS, modified Rankin Scale.

**Table 2:** Baseline characteristics stratified by 3-month functional outcome (favorable, mRS 0–2 vs. poor, mRS 3–6).

Characteristic	Favorable (*n* = 134)	Poor (*n* = 203)	*p* Value
Age, years	67 (54–73)	71 (60–80)	<0.001
Male sex, %	60.4	58.6	0.825
Admission GCS	14 (13–15)	9.0 (5.0–12.0)	<0.001
Hematoma volume, mL	6.1 (2.0–13.2)	21 (9–49)	<0.001
History of hypertension, %	63.4	70.9	0.185
Systolic BP, mmHg	178 (150–200)	189 (166–211)	0.050
Glucose, mg/dL	124 (99–161)	135 (111–171)	0.003
LDL cholesterol, mg/dL	112 (90–138)	102 (77–129)	0.004
Hemoglobin, g/dL	14 (13–15)	14 (12–15)	0.42
aPTT, s	24 (22–26)	23 (21–27)	0.694
NLR	4.4 (2.5–8.3)	5.4 (2.8–10.5)	0.083
IVH present, %	17.9	49.3	<0.001
NCCT hypodensities, %	4.5	27.6	<0.001
Island sign, %	6.7	31.0	<0.001
Swirl sign, %	0.0	10.8	<0.001

Continuous variables are median (IQR) compared with the Mann–Whitney U test; categorical variables are percentages compared with the chi-square test. GCS, Glasgow Coma Scale; IVH, intraventricular hemorrhage; LDL, low-density lipoprotein; NCCT, non-contrast computed tomography; NLR, neutrophil-to-lymphocyte ratio.

**Table 3:** Association between non-contrast CT imaging markers and poor functional outcome at 3 months.

CT Imaging Marker	Present, *n* (%)	Absent, *n* (%)	Poor Outcome (mRS 3–6), *n* (%)	Odds Ratio (95% CI)	*p* Value
Swirl sign	22 (6.5)	315 (93.5)	22 (10.8)	33.35 ^a^	<0.001
Blend sign	26 (7.7)	311 (92.3)	24 (11.8)	8.85 (2.06–38.10)	0.001
Black hole sign	36 (10.7)	301 (89.3)	34 (16.7)	13.28 (3.13–56.28)	<0.001
Island sign	72 (21.4)	265 (78.6)	63 (31.0)	6.25 (2.99–13.09)	<0.001
Hypodensities	62 (18.4)	275 (81.6)	56 (27.6)	8.13 (3.39–19.49)	<0.001
Satellite sign	14 (4.2)	323 (95.8)	12 (5.9)	4.15 (0.91–18.83)	0.087

Odds ratios (ORs) and 95% confidence intervals (CIs) were calculated using univariable logistic regression. Poor functional outcome was defined as mRS 3–6 at 3 months. ^a^ OR estimated using the Haldane–Anscombe correction owing to a zero cell count in the favorable-outcome group; the estimate is unstable and provided for descriptive purposes only. CI, confidence interval; mRS, modified Rankin Scale.

**Table 4:** Association of prognostic scores with functional outcome at 3 months.

Prognostic Score	Favorable Outcome (mRS 0–2), Median (Min–Max)	Poor Outcome (mRS 3–6), Median (Min–Max)	Odds Ratio (95% CI)	*p* Value
ICH score	1 (0–3)	2 (0–6)	4.14 (3.04–5.64)	<0.001
FUNC score	10 (7–11)	8 (1–11)	0.47 (0.39–0.57)	<0.001
Admission GCS	14 (3–15)	9 (3–15)	0.60 (0.53–0.68)	<0.001

Odds ratios and 95% confidence intervals were derived from univariable logistic regression. Poor functional outcome was defined as mRS 3–6 at 3 months. Abbreviations: FUNC, Functional Outcome in Patients with Primary Intracerebral Hemorrhage; GCS, Glasgow Coma Scale; ICH, intracerebral hemorrhage; mRS, modified Rankin Scale.

**Table 5:** Primary Firth penalized multivariable logistic regression model for poor functional outcome at 3 months.

Variable	Adjusted Odds Ratio (95% CI)	*p* Value
Admission Glasgow Coma Scale	0.66 (0.57–0.74)	<0.001
Hematoma volume (per mL)	1.04 (1.01–1.06)	0.003
Hypodensities on NCCT	3.89 (1.46–11.65)	0.010
History of hypertension	2.79 (1.37–5.95)	0.006
LDL cholesterol (per 10 mg/dL)	0.88 (0.80–0.97)	0.008

Firth penalized logistic regression on the primary model (*n* = 282; 161 poor outcomes). Odds ratios < 1 indicate lower odds of poor outcome; confidence intervals are profile penalized-likelihood intervals. The full automated backward-selection model, including additional but statistically unstable variables (aPTT, hemoglobin, and combined antithrombotic therapy), is provided as a sensitivity analysis in Table 6. Abbreviations: CI, confidence interval; LDL, low-density lipoprotein; NCCT, non-contrast computed tomography.

**Table 6:** Sensitivity analysis: full automated backward-selection multivariable model on the identical cohort (*n* = 282).

Variable	Adjusted Odds Ratio (95% CI)	*p* Value
Admission Glasgow Coma Scale	0.528 (0.431–0.646)	<0.001
Hematoma volume (per mL)	1.065 (1.026–1.105)	0.001
Hypodensities on NCCT	8.998 (2.113–38.307)	0.003
History of hypertension	3.277 (1.199–8.957)	0.021
Combined antiplatelet + anticoagulant use	22.777 (1.919–270.295)	0.013
aPTT (per second)	1.149 (1.028–1.285)	0.015
Hemoglobin (per g/dL)	1.295 (1.025–1.637)	0.031
LDL cholesterol (per mg/dL)	0.981 (0.967–0.996)	0.012

Automated backward Wald selection entering univariably significant and clinically relevant variables. This model is presented for transparency only; the combined-therapy estimate (*n* = 10) and the aPTT and hemoglobin associations are statistically unstable (see Section 3.4). The primary penalized model (Table 5) is the primary analysis.

**Table 7:** Linear regression analysis for factors affecting length of hospital stay.

Variable	B (95% CI)	SE	β	t	*p* Value	VIF
Admission mRS	0.272 (0.147–0.397)	0.063	0.355	4.308	<0.001	1.148
Hematoma volume	−0.007 (−0.013 to 0.000)	0.003	−0.175	−2.016	0.046	1.276
Swirl sign (present)	−0.653 (−1.253 to −0.053)	0.303	−0.176	−2.154	0.033	1.126
NLR	0.024 (0.000–0.048)	0.012	0.160	1.993	0.048	1.093
Epileptic seizure (present)	0.465 (0.033–0.897)	0.218	0.167	2.130	0.035	1.037

Dependent variable: length of hospital stay (natural log-transformed). Model statistics: F = 6.662; *p* < 0.001; R^2^ = 0.197; adjusted R^2^ = 0.167. A backward selection method was applied. Abbreviations: B, unstandardized coefficient; CI, confidence interval; mRS, modified Rankin Scale; NLR, neutrophil-to-lymphocyte ratio; SE, standard error; VIF, variance inflation factor; β, standardized coefficient.

## Data Availability

The datasets generated and/or analyzed during the current study are available from the corresponding author on reasonable request. The data are not publicly available due to privacy restrictions.
